# What matters to infrequent customers: a pragmatic approach to understanding perceived value and intention to revisit trendy coffee café

**DOI:** 10.1186/s40064-016-2259-5

**Published:** 2016-05-17

**Authors:** Hiram Ting, Ramayah Thurasamy

**Affiliations:** Institute of Borneo Studies, Universiti Malaysia Sarawak, Kota Samarahan, Sarawak Malaysia; Institute of Graduate Studies, SEGi University, Petaling Jaya, Selangor Malaysia; School of Management, Universiti Sains Malaysia, Georgetown, Penang Malaysia; UTM International Business School (UTM-IBS), Universiti Teknologi Malaysia, Kuala Lumpur, Wilayah Persekutuan Kuala Lumpur Malaysia

**Keywords:** Revisit intention, Perceived value, Product, Service, Experience, Quality

## Abstract

Notwithstanding the rise of trendy coffee café, little is done to investigate revisit intention towards the café in the context of developing markets. In particular, there is a lack of study which provides theoretical and practical explanation to the perceptions and behaviours of infrequent customers. Hence, the study aims to look into the subject matter by using the theory of reasoned action and social exchange theory as the underpinning basis. The framework proposed by Pine and Gilmore (Strat Leadersh 28:18–23, 2000), which asserts the importance of product quality, service quality and experience quality in a progressive manner, is used to decompose perceived value in the model so as to determine their effects on intention to revisit the café. Given the importance to gain practical insights into revisit intention of infrequent customers, pragmatism stance is assumed. Explanatory sequential mixed-method design is thus adopted whereby qualitative approach is used to confirm and complement quantitative findings. Self-administered questionnaire-based survey is first administered before personal interview is carried out at various cafés. Partial least squares structural equation modelling and content analysis are appropriated successively. In the quantitative findings, although product quality, service quality and experience quality are found to have positive effect on perceived value and revisit intention towards trendy coffee café, experience quality is found to have the greater effect than the others among the infrequent customers. The qualitative findings not only confirm their importance, but most importantly explain the favourable impressions they have at trendy coffee café based on their last in-store experience. While product and service quality might not necessary stimulate them to revisit trendy coffee café, experience quality driven by purposes of visit would likely affect their intention to revisit. As retaining customers is of utmost importance to businesses, and infrequent customers more than often make up the majority of the customers, the study provides meaningful and practical understanding of revisit intention.

## Background

Creating customer value has become an essential component that contributes to business competitive advantage (Woodruff 1997). The ability of any businesses to deliver superior value is pivotal to customers’ perception towards the quality of their offerings. The added value offered to and appreciated by customers, be it product or service, is believed to have consistently generated favourable behavioural outcomes, such as loyalty, consumption behaviour and repeated visit (Wakefield and Blodgett 1996). Hence, in food and beverage businesses, it is pivotal to know customers’ perceptions so as to come out with effective operation designs to stimulate their revisit intention.

Although trendy coffee, such as posh coffee, may have once seemed high-priced, people today consider it as an affordable and even indispensable luxury. Despite being a developing country with a population of 30 million, Malaysia is one of the emerging markets which shows promising association between Gross Domestic Product per capital and the amount of coffee consumed (International Coffee Council 2014). Since Malaysian coffee market is in the increasing trend, various business organizations are willing to invest in coffee-related businesses. As a result, the growth of retail coffee franchises, such as Starbucks, Coffee Bean & Tea Leaf, Earthlings, and Dômeand, can be seen in Malaysia in the past decade. Trendy coffee café has become one of the fastest growing business in food and beverage industry, and its businesses is estimated to grow more rapidly in the coming years (Agriculture and Agri-Food Canada 2014).

Competitions between trendy coffee cafés and conventional coffee shops are predictably inevitable and intense. It compels the managers to find cutting-edge strategy to improve their overall quality to enhance customers’ perception and their repeat consumption in store. In addition to attracting new customers, retaining existing customers becomes as important to business’s long term sustainability (Hoekstra et al. 1999). Despite the prospects of trendy coffee café business and the extant of literature on customer behaviour in food and beverage industry, it is somewhat surprising that little study has been done to delve into customer perception and revisit intention towards trendy coffee café in the context of developing markets. Since existing customers are made up by frequent and infrequent ones, past findings pertaining to revisit intention of the latter are apparently lacking. While perceived value generates transactions between providers and recipients, it is relatively unknown if the same can be said about infrequent customers. Hence, this study aims to look into the relationship between perceived value, revisit intention and their antecedents of the infrequent customers towards trendy coffee café in Malaysia. The framework proposed by Pine and Gilmore (2000), which asserts the importance of product quality, service quality and experience quality in a progressive manner, is used as antecedent variables to provide more explanation to perceived value. The theory of reasoned action (TRA) and social exchange theory (SET) are appropriated to provide theoretical basis to postulated relationships in the study. Instead of addressing theoretical gap, mixed-method approach is used to connect the underpinning theories to the context of the study so as to usher in more relevant and pragmatic discussions (Granek and Nakash 2015; Seddon and Scheepers 2011). As such the understanding of revisit intention of infrequent customers towards trendy coffee café in Malaysia would be extended. Managerial implications are highlighted to articulate the practical contribution of the study.

## Literature review

### Trendy coffee café

Trendy coffee café, which is also known as coffee house or coffee concept shop, is a place where coffee is served as the main beverage but food and other beverages are also available for consumption. Unlike conventional coffee house and local food court, it is designed to provide customers with a pleasant in-store experience. Even though each trendy coffee café has its own characteristics, they share the concept of quality and comfort (Ganea 2012). As a result, serving the best coffee is not always the only objective to satisfy customers, but also creating a coffee-drinking experience to leave them with good impression (Stark 2013). Wi-Fi connection and seating arrangements are some of the augmented products to stimulate their interest and enhance in-store experience.

The economic and social development in Malaysia as well as the increasing exposure to the western culture has seen the dynamic change of people’s values and lifestyles (Verma 2002). Although the quality of coffee, such as its ingredients and taste, used to be the prime reason for dining at a coffee café, other factors have begun to play significant part in their decision making and revisit intention. Notwithstanding a developing country, Malaysian customers are found to be prepared to pay premium price for a cup of coffee when they see additional values in it. Consequently, trendy coffee cafés, especially the retail coffee franchises, have evolved to become not only a dining place, but also a venue for personal leisure and collective socialization. It even becomes a place for teenagers to hangout (de Luca and Pegan 2013). Ever since Starbucks began its maiden business operation in the capital city of Malaysia in late nineties, trendy coffee cafés have grown rapidly and can now be seen in many populated areas, such as shopping complexes, airports, city centres and town areas throughout the country (Balakrishnan et al. 2009).

### Perceived value

Perceived value is one of the most important components in marketing and has become a driving factor to behavioural outcomes in marketplace (Hwang and Kandampully 2015). Value conception is closely associated with the perception of quality (Alex and Thomas 2011). Past findings have shown that product quality has quantifiable impact on customer intention (Woodruff 1997; Alex and Thomas 2011; Asmayadi and Hartini 2015). In the same vein, service quality and performance has long been regarded as an important ingredient that business uses to generate favourable expectation and measure satisfaction, thus ushering in locus of value creation (Parasuraman et al. 1988; Chathoth et al. 2014). Moreover, recent studies have also found that customers make transactions not only for functional reasons, but also emotional satisfaction, which includes fun or enjoyment. Such experience is found to have substantial impact on customer behavioural intention (Ali et al. 2015; Yu and Fang 2013).

The framework proposed by Pine and Gilmore (2000) demonstrates the progression of economic value and provides a comprehensive understanding between the capacity of business and customer behaviour. Apart from the offering of products, it reveals the need and importance of progression in stages: from product to service, and from service to experience. The rationale of the framework is that achieving customer expectation with product might not be sufficient for business to create value with customers in contemporary and future markets. Given the fact that trendy coffee cafés offer not only exquisite food and beverages, but also fine services, and pleasant atmosphere which enhance customers’ overall experience, it is interesting to know the perception of infrequent customers towards product quality, service quality and experience quality towards trendy coffee cafés, and how such perception affects their perception of value and revisit intention.

### Perceived product quality

Quality has been traditionally defined as a zero error rate, and that is the ability to produce a perfect product at the first try (Parasuraman et al. 1985). As far as customer perception is concerned, the superiority of the product is often regarded as the primary assessment of quality. Past studies have come out with various dimensions to define product quality (Madu et al. 1995; Eduardo et al. 2008; Garvin 1984), and contradicting views are nevertheless found. However, product quality can be generally described as the collection of characteristics and features of a product that contribute to its ability to meet or go beyond given requirement (Parasuraman and Grewal 2000; Ngoc and Uyen 2015). Therefore, perceived product quality can be described as the perceived ability of a product to provide satisfaction relative to the alternatives. It is claimed that product offering value for money not only influences customer behaviour at pre-purchase stage but also their satisfaction as well as post-purchase behaviour (Alex and Thomas 2011). This implies that when customers perceive value in the products they buy or consume due to its superior quality, they are willing to pay more for it, visit the store again and even tell others about it.

In light of the aforementioned, the conception of product quality is of relevance to the present study as it refers to customers’ perception towards the products offered by trendy coffee cafés, which in turn infers their evaluation on how well the product specification fits the expectation (Jakpar et al. 2012). Since coffee drinks are the main products and their prices are noticeably higher than those offered in conventional eateries, it is interesting to know the effect of perceived product quality on revisit intention towards trendy coffee café in a developing market.

### Perceived service quality

Similar to product quality, various descriptions have been given to explain service quality, including the widely adopted SERVQUAL model (Parasuraman et al. 1988). Service quality has been described as a function of differences between customer expectation and business services (Parasuraman et al. 1985). Customers are often found to be judging the quality of service by comparing what they want from the service with what they eventually receive (Gronroos 1983; Lau et al. 2013). Given the practical gap between what customers want and what they receive, service quality is largely accepted as the perception towards service experience (Ryu et al. 2012). The assumption behind this understanding is that customers perceive quality of service based on service performance or what they actually receive from the deliverance. This includes the behaviour of service employees which has been found to have significant effect on purchase behaviour (Abebe 2014). It has become a significant differentiator and the most powerful competitive weapon to business for attaining efficiency and business performance (Bharadwaj et al. 2015).

In any competitive environment, it is evident that a well-designed process of transaction during which service is purveyed and received is pivotal to business performance and customer satisfaction. When service is perceived as of quality, it generates positive word-of-mouth and continuous visit (Ryu et al. 2012). Since services are variable, and they are produced and consumed at the same time, service providers are often found to be under scrutiny (Kotler and Keller 2007). Hence, perceived service quality is adopted in the present study to investigate its relationship with perceived value and revisit intention of infrequent customers towards trendy coffee café.

### Perceived experience quality

The overall level of customer satisfaction and the impression a customer has through interacting with a specific product or service are understood as contextual experience (Alex and Thomas 2011). Hence, customer experience can be described as an engaging act of value co-creation between product or service providers and customers (Schmitt 2010). It can further be defined as cognitive and affective assessment in the process of direct and indirect interaction which eventually affects and moulds perception and subsequent behaviours (Meyer et al. 2007; Page and Conell 2006). Albeit related, experience differs from product and service. While a product is a tangible object, and a service is an intangible activity or process, experience is a personal attachment to the service process in the journey with a series of touch point (Johnston and Kong 2010; Poulsson and Kale 2004). This indicates that the creation of customer experience is embedded in the performance of the business ability to personalize specific prerequisite (Mascarenhas et al. 2006). By instilling memorable experience into the customers across various touch points, it reinforces higher level of loyalty and repeat purchase (Stuart 2006).

It is well documented that an effective creation of customer positive experience is essential to constructing customer loyalty, retention, and subsequently financial success (Klaus and Maklan 2012; Lemke et al. 2011). When customers are impressed in their experience, they tend to remember it and repeat their behaviours. As it is known that trendy coffee cafés offer not only products and services, but also cosy and conducive environment to facilitate various activities, such as reading, socializing and group discussions, they create and offer memorable experience for customers as well. Hence, perceived experience quality is also included in the present study to examine its effect on infrequent customers’ perception towards value and revisit intention.

### Revisit intention

Intention refers to subjective likelihood of performing a behaviour (Ajzen and Fishbein 1980). It is deemed as the direct determinants of the corresponding behaviour. Past research has supported that beliefs and attitudes are precursors of customers’ visible response, such as their purchase and repurchase intention (Huang et al. 2004; Wang et al. 2009). In like manner, past studies in food and beverage sector have also indicated that favourable perception and experience, thus overall satisfaction, are important predictors of customer intent to return (Nadiri and Gunay 2013; Oh 2000; Pettijohn et al. 1997; Qu 1997; Weiss et al. 2005). Therefore, intention is often used to better understand how perception can affect subsequent behaviour.

It is well documented that intention to purchase a product is highly related with the product quality (Wells et al. 2011). In other words, customers are willing to pay more if they perceive value in the product (Michaud et al. 2012). However, studies have also suggested the relevance of other influences, such as internal impulse and external environment (Puccinelli et al. 2009). This ushers in the importance of service quality and experience quality. It is no surprise that perceived service quality is also found to affect behavioural intention (Kim and Moon 2009). Service outcomes, such as revisit intention, come from the integration of customers’ perceived performance, expectation, and normative evaluation to the physical environment in which a service process takes place (Wakefield and Blodgett 1999). Past studies not only offer evidence that service quality perceptions positively affect behavioural intentions (Zeithaml et al. 1996), they also highlight the effect of experiential value on positive word of mouth and intention to revisit (Nadiri and Gunay 2013). Hence, revisit intention is adopted as the dependent variable or outcome construct in the study.

### Frequency of visit

Frequency of visit is found to be associated with perception towards restaurant performance (Kim and Kim 2004). It is also found to be related to loyalty to preferred restaurant and willingness to pay more for the product and service (Clark and Wood 1998). The underlying principle lies with the expectations of quality. The more frequently customers visited the restaurant, the higher their expectation on quality dimensions (Johnson and Mathews 1997). Hence, frequent customers are likely to have higher expectations of product and service quality than infrequent ones as they anticipate the deliverance of quality during their visits (Bornstein 1989). Moreover, frequent customers are described as ones who will place more reliance on information from internal sources, such as their memories, than external sources, such as advertisements and friends’ opinions than infrequent ones (Foulkes 1984). However, in a competitive business environment, the perceptions of infrequent customers cannot be overlooked and they might represent the larger segments in the market. Given the exploratory component of this part of the study, the perception of infrequent customers towards product quality, service quality and experience quality, and revisit intention towards trendy coffee café is looked into. Since past literature reviews little to define frequency of visit in the context of trendy coffee café, a preliminary study is conducted to explore and differentiate infrequent customers from the frequent ones.

### Theoretical consideration

Two theories are utilized as the basis to postulate the relationships of variables for the present study. Firstly, the theory of reasoned action (TRA), developed by Ajzen and Fishbein (1980), serves as the primary theoretical foundation to investigate the effect of perceived quality on revisit intention towards trendy coffee café. While belief is generally described as specific descriptions of the object’s attributes, attitude is defined as a learned predisposition which ushers in response in a consistent manner (Aaker et al. 2001; Fishbein 1967). As such, the perceptions towards product quality, service quality and experience quality are constructed as beliefs towards trendy coffee café, and perceived value as attitude in the study. Since TRA elucidates direct relationship between attitude and behavioural intention, it establishes the basis for the relationship between perceived value and revisit intention.

Secondly, the social exchange theory (SET) is also utilized as supporting basis to the postulated relationships. SET is among the most influential conceptual paradigms for understanding interdependent transactions (Cropanzano and Mitchell 2005). One of the most fundamental tenets is the expectation of reciprocity (Gouldner 1960). It is also known as reciprocal exchange whereby one party’s actions are contingent on the other’s behaviour (Molm 2003). Once the transaction is in motion, each outcome will create a self-reinforcing cycle. Such transaction is most often studied in workplace relationships (Shore et al. 2004), and marketplace (Houston et al. 1992; De Ruyter and Wetzels 2000). Given the fact that exchange of value is what drives the development of trendy coffee café, SET provides theoretical and practical basis to the reciprocal relationship between perceived qualities and revisit intention of infrequent customers.

### Framework and hypotheses

**Fig. 1:**
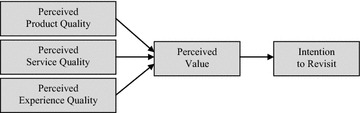
Research framework

In light of the underlying theories and literature pertaining to perceived product quality, service quality, experience quality, perceived value and revisit intention, a research framework is developed as shown in Fig. 1.

As past studies generally show the positive effect of perceived product quality, service quality and experience quality on perceived value and various behavioural outcomes (Yu and Fang 2013; Pine and Gilmore 2000), positive-directed hypotheses are developed to test the effect of the said perceptions towards trendy coffee café on revisit intention for infrequent customers. The hypotheses are formulated as follows:

H_1_: There is positive effect of perceived product quality on perceived value of infrequent customers towards trendy coffee café.
H_2_: There is positive effect of perceived service quality on perceived value of infrequent customers towards trendy coffee café.
H_3_: There is positive effect of perceived experience quality on perceived value of infrequent customers towards trendy coffee café.
H_4_: There is positive effect of perceived value on revisit intention of infrequent customers towards trendy coffee café.

## Methods

**Fig. 2:**
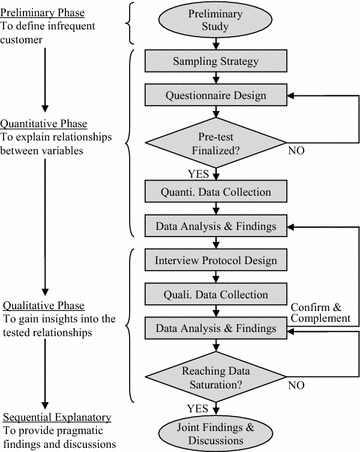
Flowchart of the research methodology

Due to the lack of literature related to perception of infrequent customers towards trendy coffee café in developing markets and also the potential deficiency of quantitative findings in providing more insights into the investigation (Creswell and Clark 2007), mixed-method design, which assumes pragmatism in its philosophical stance, was adopted in the present study (Teddlie and Tashakkori 2009). As shown in Fig. 2, sequential explanatory design is deemed the most appropriate design as it uses qualitative approach to confirm and complement the findings from the quantitative study (Venkatesh et al. 2013). The combination or “mix” of two approaches would be able to offer more pragmatic and meaningful findings and discussions, thus be of better relevance to the context of the study (Granek and Nakash 2015).

A preliminary study using qualitative interview was necessary prior to the main investigation to define infrequent customers based on frequency and purpose of visit. The interview was conducted at the trendy coffee cafés, such as Starbucks and Coffee Beans, rather than anywhere else for two reasons. Firstly, the target respondents had to be customers of any trendy coffee café. Secondly, it was more practical to interview them at the natural setting to find out why they visited trendy coffee café infrequently (Marshall 1996). As such, it is evident that purposive sampling technique was most suited to sample the respondents for the purpose of the study (Onwuegbuzie and Collins 2007). Thirty customers were approached and asked about their frequency and purpose of visit to trendy coffee cafés and sixteen of them were found to be infrequent customers. Hence, frequency of visit was included in the quantitative study as qualifying question so as to set apart the infrequent customers from the frequent ones in the dataset.

In the quantitative stage, self-administered questionnaire was used. In addition to questions about respondents’ demographic characteristics, all statements related to perceived product quality, service quality, experience quality, perceived value and revisit intention were adapted from past studies (Yu and Fang 2013; Pine and Gilmore 2000). Multiple statements were used to facilitate reliability and validity check in measurement analysis. A seven-point Likert scale was used, where 1 indicates strongly disagree to 7 indicates strongly agree in the questionnaire. After content validity was ascertained by a marketing professor, a pre-test via debriefing method was administered on three individuals to determine the usability and comprehensibility of the questionnaire so as to eliminate potential problems associated with the wordings, instructions and questionnaire design (Bazera 1996; Hunt et al. 1982).

Using purposive sampling technique, 350 copies of questionnaire were distributed at trendy coffee cafés in Malaysia. 250 usable copies were subsequently collected on the spot, with the acceptable response rate at 71 percent (Richardson 2005; Nulty 2008). Statistical Package for Social Sciences (SPSS) was then used for data entry and cleaning purpose. After setting apart data from frequent customers and removing influential outliers and cases with serious missing values, 162 cases were retained in the dataset for data analysis using structural equation modelling (SEM). The sample size was deemed adequate as it was able to detect medium effect size at a statistical power of 80 % using power analysis (Cohen 1988; Hair et al. 2014). Since the emphasis of the study is on prediction, structural equation modelling of partial least squares (PLS–SEM), which is a variance-based SEM, is regarded more appropriate in analysis (Hair et al. 2014).

Once the quantitative findings were obtained, an interview protocol was designed accordingly. The purpose was to find out why infrequent customers would or would not revisit trendy coffee café. Semi-structured interview was administered to allow probes so as to elicit more detailed information pertaining to perceived quality, value and revisit intention from the customers on the basis of data richness (Tong et al. 2007). Fifteen interviews were predetermined with reference to past literature (Guest et al. 2006), and like the preliminary study, they were conducted at various trendy coffee cafés. This was to ensure that all sampled respondents were the actual customers and they could justify their responses with subjective experiences. All interviews were audio-recorded, transcribed and analyzed using content analysis (Kurasaki 2000). Utilizing inter-coder agreement in analysis, data saturation was achieved in the tenth transcript (Carey et al. 1996; Morse 1995). Given the purpose of sequential explanatory mixed-method design, the qualitative findings would then be useful to confirm and complement the quantitative findings pertaining to revisit intention towards trendy coffee café (Onwuegbuzie and Collins 2007).

## Findings and discussions

### Preliminary study

In the preliminary study, the interview findings show that the infrequent customers are those who seldom visit trendy coffee café. Either they visit the café not more than once a month or when there is a specific purpose for them to be there. Generally, they neither take the initiative to visit nor suggest to having coffee at trendy coffee café. More than often they are asked to meet up friends there or they need a place to rest, such as at the airport or shopping complex.

### Quantitative stage

As sequential explanatory mixed-methods design is adopted, the findings are presented in quantitative stage and qualitative stage. Out of the 162 respondents, the numbers of male and female respondents are about equal. Malay and Chinese respondents make up the majority of the sample as they are the two most populous ethnic groups in Malaysia, and they are more likely to visit trendy coffee cafés which are located in urban areas. Moreover, 110 respondents who are aged 21–25 are found in the sample, showing the popularity of the café among university students and young adults.

#### Measurement model

Before moving into hypothesis testing, construct validity was first determined. Table 1 indicates the assessment of reliability and validity in the data of the study. The composite reliability (CR) and Cronbach alpha values of more than 0.70 demonstrate that these constructs possess adequate level of internal consistency (Gefen et al. 2000). Similarly, the constructs under investigation demonstrate good convergent validity as they all achieve the minimum threshold value of 0.5 for average variance extracted (AVE) (Bagozzi and Yi 1988). It explains that the items explain more than 50 % of the construct’s variances (Hair et al. 2014). Hence, no item was removed from the observation.

**Table 1:** Internal consistency and convergent validity

Construct	Item	Loading	AVE	CR
Perceived	PROD1	0.903	0.679	0.861
Product quality	PROD2	0.910		
	PROD3	0.626		
Perceived	SERV1	0.827	0.772	0.910
Service quality	SERV2	0.904		
	SERV3	0.903		
Perceived	EXP1	0.864	0.693	0.871
Experience quality	EXP2	0.831		
	EXP3	0.801		
Perceived value	VAL1	0.813	0.645	0.845
	VAL2	0.848		
	VAL3	0.745		
Revisit intention	INT1	0.858	0.762	0.906
	INT2	0.880		
	INT3	0.881		

Assessment of discriminant validity using Henseler et al.’s heterotrait-monotrait (HTMT) ratio of correlations (2015) criterion is determined as shown in Table 2. Since the correlation values corresponding to the respective constructs do not violate HTMT.90 criterion (Henseler et al. 2015), it is suffice to conclude that construct validity is established in the measurement model.

**Table 2:** HTMT criterion

	EXP	PROD	SERV	VAL	INT
EXP					
PROD	0.782				
SERV	0.852	0.832			
VAL	0.871	0.783	0.788		
INT	0.526	0.522	0.577	0.798	

*Criteria* Discriminant validity is established at HTMT_0.90_

#### Structural model

Before performing latent variable analysis in structural model, it is important to ensure that there are no collinearity issues between the constructs under investigation. Table 3 presents that the variance inflation factor (VIF) value for each construct is lower than the offending value of 3.3 (Diamantopoulos and Siguaw 2006). Hence, it suggests that collinearity is of no concern in the study.

**Table 3:** Collinearity assessment

	VAL	INT
EXP	2.084	
PROD	1.849	
SERV	2.356	
VAL		1.000

In order to test the hypotheses, bootstrapping procedure is used to generate results for each path relationship in the model as shown in Table 4. Bootstrap sub-samples with 5000 cases are created to allow the procedure to estimate the model for each subsample (Hair et al. 2014). All path relationships are found to be significant at 99 and 95 % confidence interval (PROD → VAL, ß = 0.226, p < 0.01; SERV → VAL, ß = 0.201, p < 0.05; EXP → VAL, ß = 0.394, p < 0.01; VAL → INT, ß = 0.623, p < 0.01). Given that all have positive relationship, it is surmised that the four hypotheses in the study are supported.

**Table 4:** Path co-efficient assessment

	Direct effect (ß)	Standard error	T-statistic	Decision
PROD → VAL	0.226	0.089	2.553**	Supported
SERV → VAL	0.201	0.097	2.078*	Supported
EXP → VAL	0.394	0.083	4.740**	Supported
VAL → INT	0.623	0.050	12.446**	Supported

** p < 0.01, * p < 0.05

Despite having decisions for the hypotheses, findings of co-efficient of determination (R^2^), the effect size (*f*^2^), as well as the cross-validated redundancy (Q^2^) of exogenous constructs on endogenous constructs are also presented as shown in Table 5. The values for co-efficient of determination (R^2^) of 0.388 and 0.523 suggest that the exogenous variables in this study explain 38.8 % of variances in perceived value and 52.3 % of variances in revisit intention; an indication of substantial explanatory capacity (Cohen 1988). Since the focus of PLS–SEM is on prediction, blindfolding procedure is used (Chin 1998). The Q^2^ values of 0.327 for perceived value and 0.289 for revisit intention, which are larger than 0, suggest that all exogenous variables possess predictive relevance over the endogenous variable (Fornell and Cha 1994).

**Table 5:** Determination of co-efficient (R^2^), effect size (*f* ^2^) and predictive relevance (Q^2^)

	R^2^	Q^2^	F ^2^	Size of effect
INT	0.523	0.289		
VAL	0.388	0.327	0.633	Large
PROD			0.058	Small to medium
SERV			0.036	Small to medium
EXP			0.157	Medium to large

Notwithstanding significant, *f*^2^ values disclose the importance of each exogenous construct to endogenous constructs. It is interesting that effect of perceived experience quality on perceived value (EXP, *f*^2^ = 0.157) is larger than that of perceived product quality and service quality(PROD, *f*^2^ = 0.058; SERV, *f*^2^ = 0.036) for infrequent customers. Evidently, perceived value has large effect on revisit intention towards trendy coffee café (VAL, *f*^2^ = 0.633).

#### Qualitative stage

Apart from questions pertaining to purposes or reasons to visit trendy coffee café, it has become necessary to find out if infrequent customers are indeed appreciating experience quality more than product and service quality, thus making them willing to revisit. The responses elicited from interviews not only confirm the role of perceived product quality, service quality and experience quality, they also complement the findings on the magnitude of experience quality as shown in Table 6.

**Table 6:** Perceptions of infrequent customers towards trendy coffee café

Themes	Codes	Selected quotes from transcripts
Perceived Product quality	Coffee quality Product quality Price Taste	“The coffee and food are nice but I think it is way too expensive” “I still prefer Kopi O (coffee without milk at local coffee café). I find the coffee there (trendy coffee café) good but not fragrant enough, and not suit my taste”
Perceived Service quality	Staff performance Amount of service	“The people there are nice, and they usually communicate politely”
		“They don’t really offer much service…” “I find them to be patient when I took some time to consider what to drink at one time”
Perceived Experience quality	Impression Atmosphere	“I don’t often go there but I have good impression on the soothing atmosphere”
	Purpose of usage	“It’s a nice place to meet people. I might consider having informal discussions there”
Unclassified	Convenience	“There is no Starbucks, Coffee Beans or Bing Coffee at my hometown… that’s why I can only go there once a while”
	Normative influence	“Some of my friends usually ask me to meet up there. So I go there sometimes”
	Economic condition	“Budget is tight and our economy is not doing so well… I will only go there if I have extra money”

In spite of being infrequent customers, the findings show that they believe there is generally good quality in the product and service offered at the trendy coffee café, and they also have had good in-store experience. Even though they think the products are expensive, and the coffee does not necessarily taste better than that of the local coffee shops, they accept that the products are usually good. In like manner, the overall perception about the service quality and performance is also positive. Note worthily, some do not think the staffs are actually doing much. The aforementioned could well explain why the effect size of the relationship between perceived product quality and perceived value, as well as the relationship between perceived service quality and perceived value is found to be minimal. This infers that product quality and service quality at trendy coffee café contribute little to their revisit intention. In other words, infrequent customers would not visit trendy coffee café because of the craving for coffee and the services.

Nevertheless, the findings show the magnitude of experience quality in value perception and revisit intention. Although they rarely consume coffee there, they have fond memory about their last in-store experience. Having good impressions and taking the venue of trendy coffee café into consideration for a specific purpose, such as meeting friends or having discussions there, justify why perceived experience quality carries the most effect on perceived value as shown in quantitative findings. Hence, it can be concluded that the experience quality of infrequent customers at trendy coffee café is mainly constituted by the impressions they have based on their previous visits and their attachment in various contact points. Infrequent customers are not there mainly for the product or service, rather they are there and might visit again for specific purposes which they have experienced before.

## Implications and conclusion

This study has provided some empirical insights into the revisit intention of infrequent customers towards trendy coffee café in a developing market with theoretical foundation. Both quantitative and qualitative findings support the notions that revisit intention towards trendy coffee café is affected by perceived value, which, in turn, is predicted by perceived product quality, service quality and experience quality. The findings also support what Pine and Gilmore (2000) advocate about the progression of economic value whereby experience quality is believed to be the cutting edge approach in contemporary market. In general, it is understood that experience quality helps augment the product or service, thus reinforcing customer’s revisit intention towards product or service providers (Clark and Wood 1998; Walls et al. 2011). It certainly holds true for infrequent customers as found in the present study.

In light of the findings, it is imperative that managers of trendy coffee café come out with comprehensive business strategies and practices to exemplify in-store experience quality so as to stimulate the interest of existing customers who visit the café infrequently. Over-emphasizing on product quality and frequent customers would likely lead to marketing myopia (Smith et al. 2010). The numbers of frequent and infrequent customers sampled in quantitative stage imply the latter belongs to the majority; and this could well reflect the actual situations of most food and beverage sectors in developing markets. As these markets are emerging as lucrative business regions, the managers cannot afford to lose infrequent ones. Therefore, the contemporary challenge is about keeping existing customers, and converting the infrequent ones to frequent ones by rewarding them with memorable experience when they are at the café.

The pragmatic approach using mixed-method design has indeed elucidated reasons behind the phenomenon under investigation. Such approach is gaining popularity due to its usefulness in providing meaningful and practical interpretations of findings (Granek and Nakash 2015). There are, however, caveats to the study which are worth investigating. Firstly, as different segments are more likely to have different perceptions (Clark and Wood 1998), the sample of the study, which is predominantly made up by university students and young adults, might have come short in extrapolating the findings to wider populations. From the perspectives of developing and multi-cultural country like Malaysia, factors, such as income level, generations and ethnicity, could prove to be vital in divulging more insights about revisit intention towards trendy coffee café. Secondly, the unclassified findings in qualitative stage also suggest that there are more to revisit intention towards trendy coffee café than perceptions about value and about product, service and experience quality. It is worth noting that normative influence could be crucial to improving revisit intention of infrequent customers. Thirdly, the re-specification of perceived value as mediator and the inclusion of moderators and multiple outcome variables could well extend TRA and SET, and thus provide more holistic understanding to revisit intention towards trendy coffee café.
